# The Impact of Remoteness on the Outcomes of Children With Prenatal Drug Exposure: A Population‐Based Cohort Study

**DOI:** 10.1111/jpc.70438

**Published:** 2026-05-25

**Authors:** Taylor Colligan, Ju Lee Oei, Barbara Bajuk, Lucinda Burns, Kate Lawler, Hannah Uebel, John Eastwood, Andrew Page, Evelyn Lee, Lauren Dicair, Charles Green, Michelle Dickson, Mithilesh Dronavalli

**Affiliations:** ^1^ School of Women's and Children's Health University of New South Wales Kensington New South Wales Australia; ^2^ Department of Newborn Care Royal Hospital for Women Randwick New South Wales Australia; ^3^ Drug and Alcohol Services Murrumbidgee Local Health District Wagga Wagga New South Wales Australia; ^4^ Mater Research Institute South Brisbane Queensland Australia; ^5^ Critical Care Program Sydney Children's Hospitals Network Sydney New South Wales Australia; ^6^ National Drug and Alcohol Research Centre University of New South Wales Kensington New South Wales Australia; ^7^ Department of Paediatrics Sydney Children's Hospital Sydney New South Wales Australia; ^8^ National Public Health Service Te Whatu Ora—Health New Zealand Dunedin New Zealand; ^9^ School of Population Health University of New South Wales Randwick Australia; ^10^ Department of Preventative and Social Medicine University of Otago Dunedin New Zealand; ^11^ Sydney Institute for Women, Children and Their Families Sydney Local Health District Sydney Australia; ^12^ Menzies Centre for Health Policy and Economics, School of Public Health University of Sydney Camperdown Australia; ^13^ Early Years Research Group Ingham Institute for Applied Medical Research Liverpool Australia; ^14^ Translational Health Research Institute Western Sydney University Penrith New South Wales Australia; ^15^ Centre for Social Research in Health University of New South Wales Kensington Australia; ^16^ Centre for Economic Impacts of Genomic Medicine Macquarie University North Ryde New South Wales Australia; ^17^ Independent Researcher Philadelphia Pennsylvania USA; ^18^ Alpha Maxx Healthcare Memphis Tennessee USA; ^19^ Poche Centre for Indigenous Health University of Sydney Sydney New South Wales Australia

**Keywords:** health disparate minority, health inequities, healthcare disparities, prenatal exposure delayed effects, substance‐related disorders, vulnerable populations

## Abstract

**Objective:**

Remote residents have worse health outcomes than metropolitan residents, but whether geography impacts the outcomes of children with prenatal drug exposure (PDE) is uncertain.

**Design and Main Outcome Measures:**

Linked population data was used to compare rates of death, hospitalisation, emergency department (ED) encounters and placement in Out‐of‐Home Care (OOHC) for children with (*n* = 208 492) and without (*n* = 1 607 662) smoking, alcohol or substance exposure in NSW, Australia, born between 2001 and 2020. The relationship between remoteness of residence, PDE, service utilisation and death was determined with interaction terms after adjusting for confounders (First Nations status, maternal age, maternal mental illness, social disadvantage). Outcomes are reported as adjusted incidence rate ratios with 95% confidence intervals.

**Results:**

Compared to other NSW children, children with PDE were more likely to die (2.90; 2.33–3.61), be placed in OOHC (52.43; 50.14–54.84), be hospitalised (1.22; 1.10–1.36) and attend ED (1.06; 1.03–1.09). Compared to metropolitan children with PDE, regional children were more likely to die (1.12; 0.78–1.61) but less likely to be hospitalised (0.82; 0.72–0.94), placed in OOHC (0.41; 0.37–0.44) and attend ED (0.93; 0.89–0.98). ED encounters increased up to 2.5‐fold for regional children with PDE from 2001 to 2020.

**Conclusions:**

Children with PDE in regional areas are likely to use fewer hospital resources than their metropolitan counterparts, but are more likely to die, mainly from preventable causes. Equitable access to preventative health and psychosocial support must be prioritised to improve outcomes of these children.

## Highlights

- What's known on this subject
  ○ Prenatal drug exposure (PDE) leads to greater mortality and risk of hospital and emergency department admissions in infancy and childhood. People living in regional areas face additional barriers to accessing care and therefore have worse health outcomes than metropolitan residents.
- What this study adds
  ○ Whether geography affects the health and survival of children with prenatal drug exposure (PDE), who are already vulnerable, is uncertain. This study demonstrated that regional children with PDE use fewer resources than their metropolitan counterparts but are more likely to die.
- How this study might affect research, practice or policy
  ○ Despite the Australian Government's funding to close the rural–urban health gap, disparities in maternal drug use and the outcomes of PDE are growing. This study emphasises the need to evaluate models of care to support women and children with PDE in regional and remote areas.

**Abbreviations**
95% CI: 95% confidence interval
ED: emergency department
HR: hazard ratio
ICD‐10‐AM: International Statistical Classification of Disease and Related Problems (10th Edition) Australian Modification
IRR: incidence rate ratio
NAS: Neonatal Abstinence Syndrome
NICU: neonatal intensive care unit
NSW: New South Wales
OOHC: out‐of‐home care
PDE: prenatal drug exposure

## 1 Introduction

Prenatal drug exposure (PDE) is a critical public health concern [1] firmly associated with adverse pregnancy and neonatal outcomes [2]. Infants with PDE may experience withdrawal, or Neonatal Abstinence Syndrome (NAS) [3], that has the potential to cause serious morbidities and mortality if untreated [4]. While improved recognition and treatment have reduced NAS‐related mortality [5], long‐term outcomes of this rapidly expanding population of children remain concerning [6, 7] due to the direct biological effects of exposure [2], and adverse socioeconomic [5, 8, 9, 10] and environmental [11] circumstances accompanying parental drug use.

We, and others, have shown that PDE and NAS increase the risk of death, hospitalisations and emergency department (ED) admissions in infancy [7, 12, 13] and until childhood [5, 6, 9, 14, 15]. Long‐term support is crucial to reduce adverse outcomes, but this is challenging in marginalised and remote communities with poor access to follow‐up services [5, 9, 16, 17] that are needed for sustained benefit [18].

PDE is increasingly affecting remote, resource‐deprived regions at alarming rates [16], especially in countries like Australia [19] and the United States [20], where the increase in rural rates of NAS was 2–2.5 times higher than in metropolitan areas [16]. Residents in remote areas face disproportionately more barriers to accessing health care services that are paramount to preventing PDE and improving outcomes, including antenatal care [5, 9] and treatment for drug use disorders [16, 17] that necessitate in‐person attendance. Furthermore, people in nonmetropolitan areas are more likely to be financially disadvantaged [21] and have limited health literacy [22, 23]. Given the high proportion of First Nations people living in regional and remote areas, anti‐Indigenous discrimination and poor accessibility to culturally sensitive healthcare also contribute to these disparities [24, 25, 26].

The impact of geographical remoteness on PDE‐related childhood outcomes remains unclear. A retrospective cohort study found that among infants with NAS, rural residence was associated with increased utilisation of outpatient and ED services in the first year of life [7]. Due to difficulties in tracking this large and often marginalised group of children, there remains little information on long‐term outcomes, particularly for those without a NAS diagnosis [27, 28]. No study has evaluated geographical disparities in childhood outcomes after PDE, despite funding initiatives from many governments, including Australia [29], to improve health outcomes in regional and remote areas.

This study used linked population data to assess whether geographical remoteness affected the long‐term health outcomes of infants with PDE, hypothesising that those in regional and remote areas would have reduced access to health services (e.g., hospitalisation, ED presentations) and higher risks of adverse outcomes, including out‐of‐home care (OOHC) placement and death.

## 2 Methodology

### 2.1 Study Design and Setting

This population birth cohort study used routinely collected birth, hospitalisation, ED, death and OOHC records of all infants born in NSW, Australia, between 1 July 2001 and 31 December 2020.

### 2.2 Record Linkage

The Centre for Health Record Linkage (CHeReL) is an independent facility responsible for the linkage of patient records for research and other purposes [30]. Extracts of patient records were linked using probabilistic methods according to names, dates of birth, addresses and hospital identification numbers, which carry a small risk of incorrect linkages (approximately 0.5%) [30]. Each patient was assigned a unique Project‐specific Person Number (PPN), ensuring complete anonymity to researchers. Details of the linked datasets are provided in Table S1.

### 2.3 Participant Selection and Grouping

**FIGURE 1:**
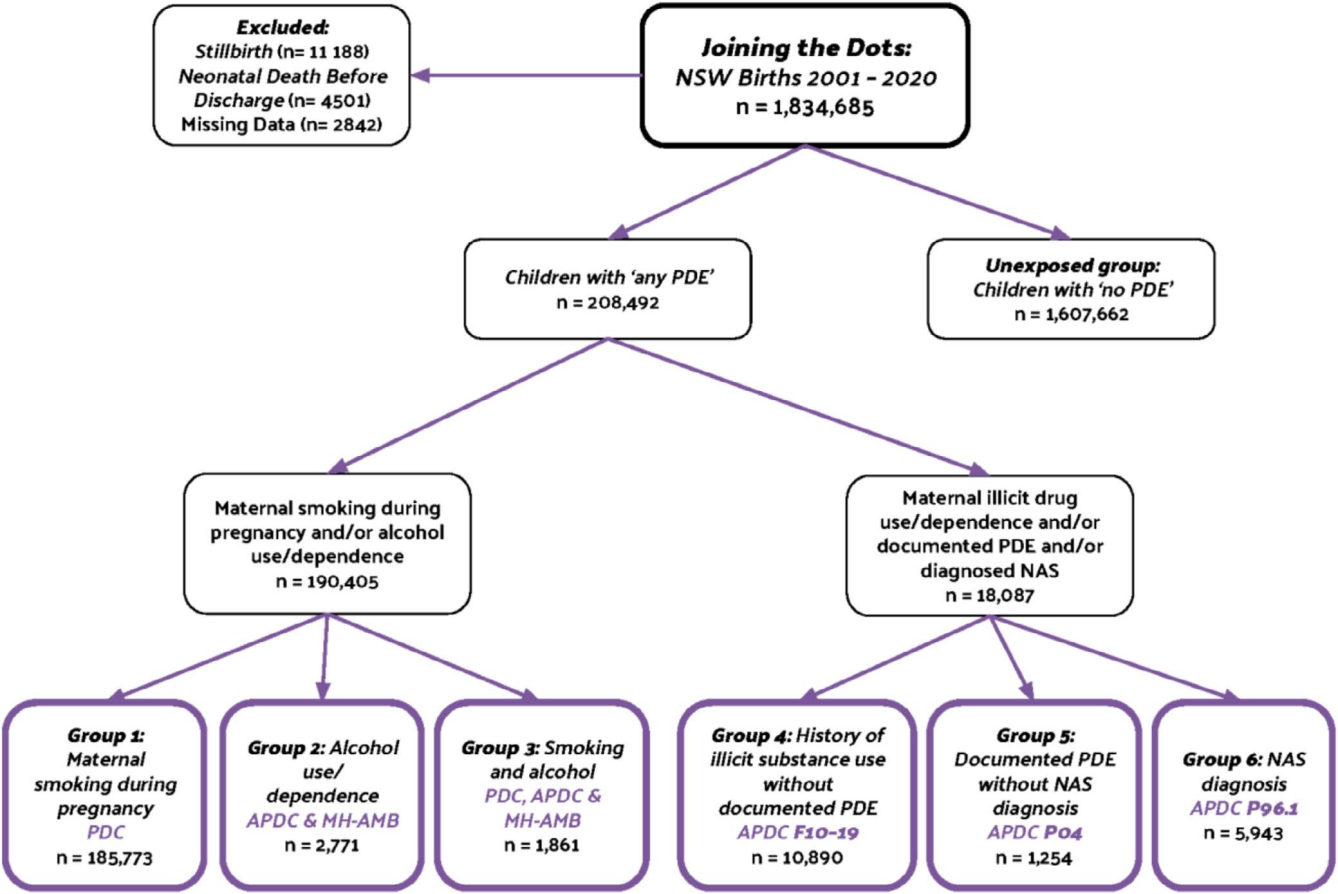
Participant flow diagram.

As outlined in Figure 1, all births in NSW (*n* = 1 834 685) were grouped based on a history of PDE after exclusion of stillbirths (*n* = 11 188), neonatal deaths before discharge (*n* = 4501) and babies with missing birth discharge data (*n* = 2842). Infants with PDE (*n* = 208 492) were grouped according to the type of substance exposure identified in maternal records and the presence of PDE or NAS diagnoses in infant records.

- Unexposed Reference Group: No Known History of SUP, PDE or NAS From Infant or Maternal Records (*n* = 1 607 662)
- A history of substance use in pregnancy (SUP) or PDE (*n* = 208 492), including
  1. Maternal smoking only during pregnancy (*n* = 185 773)
  2. Maternal alcohol use/dependence only (*n* = 2771)
  3. Maternal smoking and alcohol use/dependence (*n* = 1861)
  4. Maternal drug use/dependence with/without smoking and/or alcohol use/dependence, but PDE not documented (ICD‐10‐AM F10–19; *n* = 10 890)
  5. Child with documented PDE, but no diagnosis of NAS at birth (ICD‐10‐AM P04.4, *n* = 1254)
  6. Child diagnosed with NAS at birth (ICD‐10‐AM P96.1, *n* = 5943)

Children with PDE were also divided into larger groups, including children exposed to tobacco smoke and/or alcohol (*n* = 190 405), and children exposed to illicit substances with or without diagnosed PDE or NAS (*n* = 18 087).

### 2.4 Definition of Remoteness

Remoteness was categorised according to the Accessibility/Remoteness Index of Australia (ARIA+), which consists of five categories stratifying relative geographic access to service centres, measured by road distance: major city (Category 0), inner regional (Category 1), outer regional (Category 2), remote (Category 3) and very remote areas (Category 4) [31]. Remoteness was also divided into larger categories for some analyses: Metropolitan (ARIA+: 0), Regional (ARIA+: 1 and 2) and Remote (ARIA+: 3 and 4).

### 2.5 Data Analysis

The effect of remoteness (ARIA+), social disadvantage (Socio‐Economic Indexes for Areas [SEIFA]), First Nations Status, maternal mental illness and maternal age on the incidence of PDE was determined by Poisson regression to calculate the incidence rate ratio (IRR). Poisson regression was also used to assess the effect of PDE on infant demographics, including prematurity, low birth weight, admission to the NICU or special care nursery (SCN), as well as outcomes beyond discharge such as death (at any age, ≤ 28 days, 29–365 days, 1–5 years, 6–10 years, 11–20 years) and placement into OOHC (at any age, ≤ 6 months and ≤ 12 months). Linear regression was used to compare birth admission length, and Cox regression was used to assess time to death, with hazard ratios (HRs) calculated. Because a large proportion of participants had no further admissions beyond birth, a zero‐inflated Poisson regression was used to compare the number of hospital and ED encounters across age groups. Negative binomial regression was used for total days in hospital and total cost of hospitalisations due to overdispersion.

Choropleth maps of NSW, divided into Statistical Areas Level 2 (SA2s) [31] were constructed to assess temporal and geographical trends within each PDE group. Within each SA2, Poisson regression was used to estimate the crude annual change in the incidence of PDE and in cohort outcomes, including death, OOHC placements, hospitalisations and ED encounters. The resultant IRRs were then merged with the dataset containing polygon coordinates for the SA2s, and maps were created using the SPMAP [32] feature in Stata 18.0. Each area was coloured according to the IRR values, with blue indicating a decrease in the rate over the study period, red indicating an increase and white indicating insufficient data for regression.

The interaction between remoteness and PDE in influencing clinical outcomes was evaluated using Poisson regression. Interactions were synergistic (IRR > 1) when the combined effect of remoteness and PDE surpassed the sum of each independently, and antagonistic (IRR < 1) when the combined effect was less than the sum.

Adjustment for confounders was based on the directed acyclic graph depicted in Figure S1, including First Nations Status, remoteness (ARIA+), social disadvantage (SEIFA), young maternal age (under 20 years), serious mental illness in the mother and calendar year of birth.

Data were presented primarily as percentages (%), adjusted IRR (aIRR) with 95% confidence interval (CI) and median (interquartile range [IQR]) for skewed data or mean (standard deviation [SD]) for normally distributed data.

All statistical analyses were performed using Stata (Basic edition; Version 18.0, College Station, TX: StataCorp LLC).

## 3 Ethics Statement

Authorised by NSW Human Research Ethics Committee on 29/06/2020 (2019/ETH12716) and the Aboriginal Health and Medical Research Council (AHMRC; 19 July 2022), 2019/ETH 12716. Personnel amendment authorised on 24/02/2023. Manuscripts were approved by the Department of Communities and Justice NSW and the AHMRC before submission.

## 4 Results

### 4.1 Maternal and Infant Demographics

Mothers with SUP were more likely to be younger, of First Nations background, have a mental health disorder, or belong to the lowest SEIFA quintiles (Table 1). Of all children exposed to substances, only 2.85% received a NAS diagnosis, with rates higher in metropolitan (3.56%) than remote areas (0.40%). Infants with any substance exposure were more likely to be premature and have low birth weight (Table 2). Those with documented PDE or NAS were more likely to be admitted to a SCN or NICU (Group 5: 4.63; 95% CI 4.30–4.99; Group 6: 4.73; 95% CI 4.57–4.89) and required longer birth admissions.

**TABLE 1:** Maternal characteristics, by type of SUP and documentation of PDE or NAS.

Characteristic		Unexposed group^a^ (*n* = 1 607 662)	Group 1: Maternal smoking during pregnancy (*n* = 185 773)		Group 2: Maternal alcohol use/dependence (*n* = 2771)		Group 3: Maternal smoking during pregnancy and maternal alcohol use/dependence (*n* = 1861)		Group 4: Maternal drug use/dependence±smoking and alcohol use/dependence, but no documented PDE (*n* = 10 890)		Group 5: Infant with PDE (ICD‐10‐AM P04.4) but not NAS (*n* = 1254)		Group 6: Infant with NAS (ICD‐10‐AM P96.1) (*n* = 5943)	
		*n* (%)	*n* (%)	aIRR^c^, ^d^ (95% CI)	*n* (%)	aIRR^c^, ^d^ (95% CI)	*n* (%)	aIRR^c^, ^d^ (95% CI)	*n* (%)	aIRR^c^, ^d^ (95% CI)	*n* (%)	aIRR^c^, ^d^ (95% CI)	*n* (%)	aIRR^c^, ^d^ (95% CI)
Young mother (< 20 years at delivery)		33 789 (2.10)	16 696 (8.99)	2.11 (2.07–2.14)	83 (3.00)	1.01 (0.81–1.26)	131 (7.04)	1.31 (1.09–1.57 = 8)	1216 (11.17)	2.46 (2.31–2.62)	92 (7.34)	1.44 (1.16–1.80)	256 (4.31)	0.83 (0.73–0.95)
History of serious mental illness^b^		19 308 (1.18)	3612 (1.94)	1.30 (1.26–1.35)	651 (23.49)	21.05 (19.27–23.00)	265 (14.24)	10.64 (9.33–12.13)	1494 (13.72)	10.04 (9.50–10.61)	69 (5.50)	3.64 (2.85–4.64)	387 (6.51)	4.40 (3.97–4.89)
Aboriginal and/or Torres Strait Islander heritage		33 266 (2.07)	25 727 (13.85)	4.45 (4.40–4.51)	147 (5.30)	1.55 (1.31–1.83)	399 (21.44)	7.56 (6.77–8.45)	2164 (19.87)	6.87 (6.56–7.20)	303 (24.16)	8.83 (7.76–10.05)	1249 (21.02)	7.37 (6.93–7.85)
SEIFA quintile^e^	1	262 355 (16.51)	35 678 (19.43)	2.24 (2.20–2.29)	287 (10.42)	0.47 (0.41–0.54)	348 (18.93)	2.16 (1.76–2.65)	1949 (18.04)	2.34 (2.15–2.54)	254 (20.45)	2.98 (2.35–3.78)	1460 (24.83)	4.20 (3.75–4.70)
	2	473 146 (29.78)	75 607 (41.18)	2.45 (2.41–2.50)	834 (30.28)	0.74 (0.67–0.83)	778 (42.33)	2.62 (2.17–3.16)	4442 (41.13)	2.76 (2.55–2.97)	443 (35.67)	2.84 (2.27–3.56)	2177 (37.03)	3.33 (2.99–3.72)
	3	269 955 (16.99)	35 836 (19.52)	2.12 (2.08–2.16)	451 (16.38)	0.71 (0.63–0.81)	344 (18.72)	2.09 (1.70–2.56)	2202 (20.39)	2.43 (2.24–2.63)	279 (22.46)	3.14 (2.48–3.98)	1178 (20.04)	3.14 (2.80–3.53)
	4	273 556 (17.22)	22 234 (12.11)	1.61 (1.58–1.65)	555 (20.15)	0.95 (0.85–1.06)	235 (12.79)	1.80 (1.46–2.23)	1390 (12.87)	1.79 (1.64–1.95)	172 (13.85)	2.00 (1.56–2.57)	676 (11.50)	1.88 (1.66–2.13)
	5	309 990 (19.51)	14 257 (7.76)	—	627 (22.77)	—	133 (7.24)	—	818 (7.57)	—	94 (7.57)	—	388 (6.60)	—
ARIA+ category^f^	0	1 273 067 (80.60)	108 601 (59.36)	—	2094 (76.28)	—	1035 (56.31)	—	6957 (64.62)	—	952 (76.84)	—	4411 (75.50)	—
	1	230 052 (14.57)	51 249 (28.01)	2.03 (2.00–2.05)	510 (18.58)	1.27 (1.15–1.40)	567 (30.85)	2.35 (2.12–2.61)	2887 (26.82)	1.75 (1.67–1.83)	231 (18.64)	1.02 (0.88–1.18)	1200 (20.54)	1.15 (1.07–1.22)
	2	69 736 (4.42)	20 328 (11.11)	2.36 (2.32–2.40)	124 (4.52)	1.06 (0.88–1.28)	180 (9.79)	2.20 (1.87–2.59)	824 (7.65)	1.48 (1.38–1.60)	45 (3.63)	0.59 (0.44–0.80)	219 (3.75)	0.58 (0.51–0.67)
	3	5295 (0.34)	1957 (1.07)	2.78 (2.66–2.91)	7 (0.26)	0.82 (0.39–1.73)	24 (1.31)	3.65 (2.43–5.48)	64 (0.59)	1.43 (1.12–1.83)	5 (0.40)	0.80 (0.33–1.93)	4 (0.07)	0.12 (0.05–0.33)
	4	1296 (0.08)	816 (0.45)	4.04 (3.77–4.33)	10 (0.36)	4.89 (2.60–9.17)	32 (1.74)	17.73 (12.32–25.52)	34 (0.32)	2.75 (1.96–3.86)	6 (0.48)	3.17 (1.41–7.13)	8 (0.14)	0.74 (0.37–1.49)

*Note:* Data expressed as *n* (%), unless otherwise stated.

Abbreviations: 95% CI, 95% confidence interval; aIRR, adjusted incidence rate ratio; ARIA+, Accessibility/Remoteness Index of Australia (measure of remoteness based on road distances to service centres); ICD‐10‐AM, International Classification of Diseases, Tenth Revision, Australian Modification; NAS, Neonatal Abstinence Syndrome; PDE, prenatal drug exposure; SEIFA, Socio‐Economic Indexes for Areas (a measure of social disadvantage in a small contained geographical area); SUP, substance use in pregnancy.

^a^ Children with no maternal smoking during pregnancy, no maternal alcohol use/dependence, no maternal drug use/dependence, no PDE and no NAS.

^b^ Recorded in the last hospital admission or episode of mental health care in an ambulatory care setting prior to birth.

^c^ Risk of PDE compared to mothers over 20 years of age, those without a history of serious mental illness, those who are.

^d^ Adjustment for young mother: Aboriginal and/or Torres Strait Islander mother, SEIFA, ARIA+; adjustment for serious mental illness in the mother: aboriginal and/or Torres Strait Islander mother, SEIFA, ARIA+, young mother (aged < 20 years); adjustment for SEIFA: ARIA+; adjustment for ARIA+: SEIFA.

^e^ 5th SEIFA quintile was defined as the base level for regression.

^f^ ARIA+ categories: Major Cities (0), Inner Regional (1), Outer Regional (2), Remote (3), Very Remote (4); ARIA+ Category 0 was defined as the base level for regression.

**TABLE 2:** Infant characteristics and outcomes, by type of SUP and documentation of PDE or NAS.

Level of care required at birth	Unexposed group^a^ (*n* = 1 607 662)	Group 1: Maternal smoking during pregnancy (*n* = 185 773)		Group 2: Maternal alcohol use/dependence (*n* = 2771)		Group 3: Maternal smoking during pregnancy and maternal alcohol use/dependence (*n* = 1861)		Group 4: Maternal drug use/dependence±smoking and alcohol use/dependence, but no documented PDE (*n* = 10 890)		Group 5: Infant with PDE (ICD‐10‐AM P04.4) but not NAS (*n* = 1254)		Group 6: Infant with NAS (ICD‐10‐AM P96.1) (*n* = 5943)	
	*n* (%)	*n* (%)	aIRR^b^, ^c^ (95% CI)	*n* (%)	aIRR^b^, ^c^ (95% CI)	*n* (%)	aIRR^b^, ^c^ (95% CI)	*n* (%)	aIRR^b^, ^c^ (95% CI)	*n* (%)	aIRR^b^, ^c^ (95% CI)	*n* (%)	aIRR^b^, ^c^ (95% CI)
Prematurity (< 37 weeks' gestation)	101.49 (6.29)	17 305 (9.32)	1.44 (1.42–1.47)	229 (8.26)	1.26 (1.11–1.44)	258 (13.86)	2.07 (1.83–2.34)	1820 (16.71)	2.49 (2.37–2.61)	325 (25.92)	3.80 (3.41–4.24)	1284 (21.61)	3.18 (3.01–3.37)
Low birth weight (< 2500 g)	80 986 (5.04)	18 770 (10.10)	1.97 (1.93–2.00)	207 (7.47)	1.47 (1.28–1.68)	303 (16.28)	3.12 (2.79–3.50)	1952 (17.92)	3.35 (3.20–3.51)	424 (33.81)	6.19 (5.63–6.82)	1427 (24.01)	4.43 (4.20–4.67)
Admission to a NICU or SCN	172 850 (14.69)	27 676 (18.50)	1.21 (1.19–1.22)	391 (16.66)	1.07 (0.97–1.18)	389 (24.31)	1.50 (1.35–1.66)	2650 (29.58)	1.85 (1.78–1.92)	728 (73.39)	4.63 (4.30–4.99)	3589 (75.04)	4.73 (4.57–4.89)

Length of birth admission	Median (IQR)	Median (IQR)	aβ^b^, ^c^ (95% CI)	Median (IQR)	aβ^b^, ^c^ (95% CI)	Median (IQR)	aβ^b^, ^c^ (95% CI)	Median (IQR)	aβ^b^, ^c^ (95% CI)	Median (IQR)	aβ^b^, ^c^ (95% CI)	Median (IQR)	aβ^b^, ^c^ (95% CI)
Median length of stay	3 (2–4)	2 (1–4)	−0.28 (−0.31 to −0.25)	3 (2–5)	0.32 (0.09–0.56)	3 (2–4)	0.50 (0.21–0.79)	3 (2–5)	1.21 (1.10–1.33)	6 (4–10)	5.22 (4.87–5.57)	9 (6–17)	9.23 (9.07–9.39)

Death	*n* (%)	*n* (%)	aIRR^b^, ^c^ (95% CI)	*n* (%)	aIRR^b^, ^c^ (95% CI)	*n* (%)	aIRR^b^, ^c^ (95% CI)	*n* (%)	aIRR^b^, ^c^ (95% CI)	*n* (%)	aIRR^b^, ^c^ (95% CI)	*n* (%)	aIRR^b^, ^c^ (95% CI)
Any age	3009 (0.19)	864 (0.47)	1.94 (1.79–2.10)	12 (0.43)	1.87 (1.06–3.30)	21 (1.13)	4.03 (2.62–6.21)	68 (0.62)	2.37 (1.85–3.03)	12 (0.96)	4.04 (2.29–7.14)	60 (1.01)	4.12 (3.22–5.41)
< 28 days	678 (0.04)	171 (0.09)	1.85 (1.55–2.22)	1 (0.04)	0.79 (0.11–5.66)	4 (0.21)	3.88 (1.44–10.45)	15 (0.14)	2.66 (1.58–4.48)	1 (0.08)	1.58 (0.22–10.23)	5 (0.08)	1.68 (0.70–4.07)
1 month to 1 year	1006 (0.06)	393 (0.21)	2.75 (2.42–3.11)	1 (0.04)	0.49 (0.07–3.49)	14 (0.75)	8.64 (5.06–14.74)	26 (0.24)	2.71 (1.81–4.06)	7 (0.56)	7.08 (3.36–14.92)	35 (0.59)	7.28 (5.15–10.30)
1–5 years	775 (0.05)	199 (0.11)	1.72 (1.46–2.03)	5 (0.18)	2.96 (1.22–7.17)	2 (0.11)	1.47 (0.37–5.92)	16 (0.15)	2.20 (1.33–3.64)	1 (0.08)	1.32 (0.19–9.39)	10 (0.17)	2.77 (1.48–5.19)
6–10 years	267 (0.02)	43 (0.02)	0.97 (0.69–1.36)	3 (0.11)	5.36 (1.70–16.91)	1 (0.05)	2.06 (0.29–14.81)	5 (0.05)	1.93 (0.79–4.73)	2 (0.16)	7.62 (1.88–30.82)	5 (0.08)	4.00 (1.64–9.77)
11–20 years	283 (0.02)	58 (0.03)	1.11 (0.82–1.48)	2 (0.07)	2.17 (0.53–8.85)	0 (0.00)	–^e^	6 (0.06)	1.69 (0.74–3.85)	1 (0.08)	2.99 (0.42–21.39)	5 (0.08)	2.99 (1.23–7.31)

Survival analysis	–	aHR (95% CI)	aHR (95% CI)	aHR (95% CI)	aHR (95% CI)	aHR (95% CI)	aHR (95% CI)
Time to death	—	1.95 (1.79–2.11)	1.87 (1.06–3.30)	4.08 (2.65–6.28)	2.38 (1.86–3.04)	4.06 (2.30–7.17)	4.19 (3.23–5.43)

OOHC	*n* (%)	*n* (%)	aIRR^b^, ^c^ (95% CI)	*n* (%)	aIRR^b^, ^c^ (95% CI)	*n* (%)	aIRR^b^, ^c^ (95% CI)	*n* (%)	aIRR^b^, ^c^ (95% CI)	*n* (%)	aIRR^b^, ^c^ (95% CI)	*n* (%)	aIRR^b^, ^c^ (95% CI)
Any age	7056 (0.44)	11 493 (6.19)	10.13 (9.82–10.46)	146 (5.27)	9.25 (7.84–10.92)	405 (21.76)	29.28 (26.44–32.43)	2480 (22.77)	30.19 (28.76–31.70)	487 (38.84)	55.73 (50.76–61.18)	2369 (39.86)	59.15 (56.34–62.11)
< 6 months	1524 (0.09)	2351 (1.27)	11.44 (10.68–12.25)	24 (0.87)	7.87 (5.25–11.80)	91 (4.89)	39.11 (31.48–48.58)	566 (5.20)	36.97 (33.35–40.98)	244 (19.46)	139.77 (121.67–160.56)	1206 (20.29)	153.96 (141.98–166.94)
< 12 months	1994 (0.12)	3146 (1.69)	11.47 (10.80–12.17)	33 (1.19)	8.03 (5.65–11.41)	122 (6.56)	39.48 (32.74–47.60)	737 (6.77)	36.26 (33.13–39.69)	272 (21.69)	118.16 (103.77–134.54)	1359 (22.87)	132.05 (122.67–142.14)

Measure of healthcare utilisation		Median (IQR)	Median (IQR)	aIRR^b^, ^c^ (95% CI)	Median (IQR)	aIRR^b^, ^c^ (95% CI)	Median (IQR)	aIRR^b^, ^c^ (95% CI)	Median (IQR)	aIRR^b^, ^c^ (95% CI)	Median (IQR)	aIRR^b^, ^c^ (95% CI)	Median (IQR)	aIRR^b^, ^c^ (95% CI)
Hospital Ad.	0–1 year	0 (0–0)	0 (0–1)	1.19 (1.17–1.21)	0 (0–0)	0.96 (0.88–1.06)	0 (0–1)	1.33 (1.19–1.49)	0 (0–1)	1.12 (1.06–1.19)	0 (0–1)	1.44 (1.28–1.63)	0 (0–1)	1.34 (1.26–1.42)
	1–5 years	0 (0–1)	0 (0–1)	1.06 (1.04–1.09)	0 (0–1)	0.94 (0.86–1.03)	0 (0–1)	1.00 (0.89–0.12)	0 (0–1)	1.12 (0.98–1.27)	0 (0–1)	1.10 (0.94–1.28)	0 (0–1)	1.22 (1.09–1.36)
	6–10 years	0 (0–0)	0 (0–0)	1.11 (1.05–1.18)	0 (0–0)	0.98 (0.77–1.24)	0 (0–0)	1.20 (0.81–1.77)	0 (0–0)	1.24 (0.96–1.60)	0 (0–0)	1.23 (0.68–2.23)	0 (0–1)	1.37 (1.03–1.84)
	11–15 years	0 (0–0)	0 (0–0)	1.08 (1.01–1.15)	0 (0–0)	1.03 (0.83–1.26)	0 (0–0)	0.70 (0.58–0.85)	0 (0–0)	1.22 (0.99–1.49)	0 (0–1)	0.88 (0.70–1.12)	0 (0–1)	1.58 (1.21–2.06)
ED Ad.	0–1 year	0 (0–1)	0 (0–1)	1.23 (1.22–1.24)	0 (0–1)	1.10 (1.01–1.20)	0 (0–1)	1.30 (1.18–1.42)	0 (0–1)	1.15 (1.11–1.20)	0 (0–1)	1.04 (0.94–1.16)	0 (0–1)	1.22 (1.16–1.29)
	1–5 years	1 (0–3)	1 (0–3)	1.13 (1.12–1.14)	1 (0–3)	1.02 (0.96–1.08)	1 (0–3)	1.10 (1.03–1.18)	1 (0–3)	1.04 (1.01–1.07)	1 (0–3)	1.02 (0.93–1.12)	1 (0–3)	1.05 (1.01–1.10)
	6–10 years	0 (0–1)	1 (0–2)	1.15 (1.14–1.17)	1 (0–2)	1.10 (1.02–1.19)	1 (0–2)	1.20 (1.10–1.32)	1 (0–2)	1.15 (1.10–1.20)	1 (0–2)	0.94 (0.83–1.06)	0 (0–2)	1.06 (0.99–1.15)
	11–15 years	0 (0–2)	1 (0–3)	1.31 (1.29–1.34)	1 (0–2)	1.23 (1.10–1.37)	1 (0–2)	1.22 (1.06–1.41)	1 (0–3)	1.44 (1.33–1.57)	1 (0–3)	1.75 (1.21–2.55)	1 (0–3)	1.93 (1.64–2.29)
LOS	0–1 year	4 (2–5)	3 (2–5)	1.03 (1.03–1.04)	4 (2–5)	1.07 (1.03–1.10)	3 (2–6)	1.28 (1.23–1.34)	4 (2–7)	1.47 (1.44–1.49)	7 (5–14)	2.68 (2.55–2.81)	11 (7–22)	3.36 (3.28–3.43)
	1–5 years	0 (0–0)	0 (0–1)	1.15 (1.13–1.17)	0 (0–1)	0.95 (0.84–1.07)	0 (0–1)	1.10 (0.95–1.27)	0 (0–1)	1.25 (1.18–1.34)	0 (0–1)	1.50 (1.24–1.82)	0 (0–1)	1.41 (1.29–1.53)
	6–10 years	0 (0–0)	0 (0–0)	1.16 (1.13–1.19)	0 (0–0)	1.05 (0.86–1.30)	0 (0–0)	1.12 (0.88–1.43)	0 (0–0)	1.29 (1.16–1.44)	0 (0–0)	1.36 (0.97–1.90)	0 (0–0)	1.44 (1.24–1.67)
	11–15 years	0 (0–0)	0 (0–0)	1.25 (1.19–1.32)	0 (0–0)	1.80 (1.26–2.58)	0 (0–0)	0.79 (0.51–1.21)	0 (0–0)	2.04 (1.69–2.75)	0 (0–0)	1.53 (0.86–2.75)	0 (0–0)	2.03 (1.56–2.64)
Cost^d^	0–1 year	3570.75 (3570.75–6171.39)	3570.75 (3570.75–6488.72)	1.22 (1.21–1.23)	3570.75 (3570.75–6171.39)	1.13 (1.08–1.18)	3570.75 (3570.75–7675.20)	1.38 (1.30–1.46)	3570.75 (3570.75–8435.05)	1.49 (1.46–1.53)	6171.39 (3570.75–13617.08)	2.35 (2.20–2.52)	11598.36 (9335.07–18408.68)	2.65 (2.56–2.73)
	1–5 years	0 (0–2595.32)	0 (0–3028.12)	1.12 (1.09–1.15)	0 (0–3028.12)	0.97 (0.79–1.20)	0 (0–3028.12)	1.08 (0.84–1.39)	0 (0–3028.12)	1.21 (1.09–1.35)	0 (0–3608.68)	1.41 (1.02–1.96)	0 (0–3520.08)	1.34 (1.15–1.55)
	6–10 years	0 (0–0)	0 (0–0)	1.14 (1.09–1.20)	0 (0–0)	1.01 (0.71–1.43)	0 (0–0)	1.21 (0.80–1.83)	0 (0–0)	1.32 (1.10–1.58)	0 (0–0)	1.36 (0.77–2.41)	0 (0–0)	1.49 (1.15–1.92)
	11–15 years	0 (0–0)	0 (0–0)	1.27 (1.17–1.39)	0 (0–0)	1.39 (0.76–2.53)	0 (0–0)	0.99 (0.49–1.99)	0 (0–0)	1.62 (1.18–2.21)	0 (0–2282.40)	1.64 (0.62–4.32)	0 (0–0)	2.06 (1.33–3.18)

*Note:* Data expressed as *n* (%), unless otherwise stated.

Abbreviations: 95% CI, 95% confidence interval; Ad., admissions; aIRR, adjusted incidence rate ratio; aβ, adjusted regression coefficient.; ED, emergency department; HR, hazard ratio; ICD‐10‐AM, International Classification of Diseases, Tenth Revision, Australian Modification; IQR, interquartile range; LOS, length of stay; NAS, Neonatal Abstinence Syndrome; NICU, neonatal intensive care unit; OOHC, out‐of‐home care; PDE, prenatal drug exposure; SCN, special care nursery; SUP, substance use in pregnancy.

^a^ Children with no maternal smoking during pregnancy, no maternal alcohol use/dependence, no maternal drug use/dependence, no PDE and no NAS.

^b^ Risk compared to Unexposed Group (children with no maternal smoking during pregnancy, no maternal alcohol use/dependence, no maternal drug use/dependence, no PDE and no NAS).

^c^ Adjusted for: aboriginal and/or Torres Strait Islander mother; serious mental illness in the mother; Accessibility/Remoteness Index of Australia (ARIA+) (measure of remoteness based on road distances to service centres); Socio‐Economic Indexes for Areas (SEIFA) (a measure of social disadvantage in a small contained geographical area); young mother (aged < 20 years); calendar year.

^d^ Inpatient hospital cost for each admission was determined according to the Australian Refined Diagnosis Related Group (AR‐DRG) cost.

^e^ Not calculable, very low death rate.

### 4.2 Geographical Distribution of PDE

Maternal smoking and/or alcohol use significantly increased with level of remoteness (Group 3: aIRR 17.73; 95% CI 12.32–25.52). Illicit substance exposure without documented PDE increased in all regional and remote areas, peaking in very remote areas (Group 4: aIRR 2.75; 95% CI 1.96–3.86). Documented PDE also peaked in very remote NSW; however, it decreased in outer regional and remote areas (Group 5 ARIA+2: aIRR 0.59; 95% CI 0.44–0.80). NAS increased slightly in inner regional NSW and decreased with remoteness, most notably in remote areas (Group 6 ARIA+3: aIRR 0.12; 95% CI 0.05–0.33) (Table 1).

### 4.3 Temporal Change in Geographical Distribution of PDE

**FIGURE 2:**
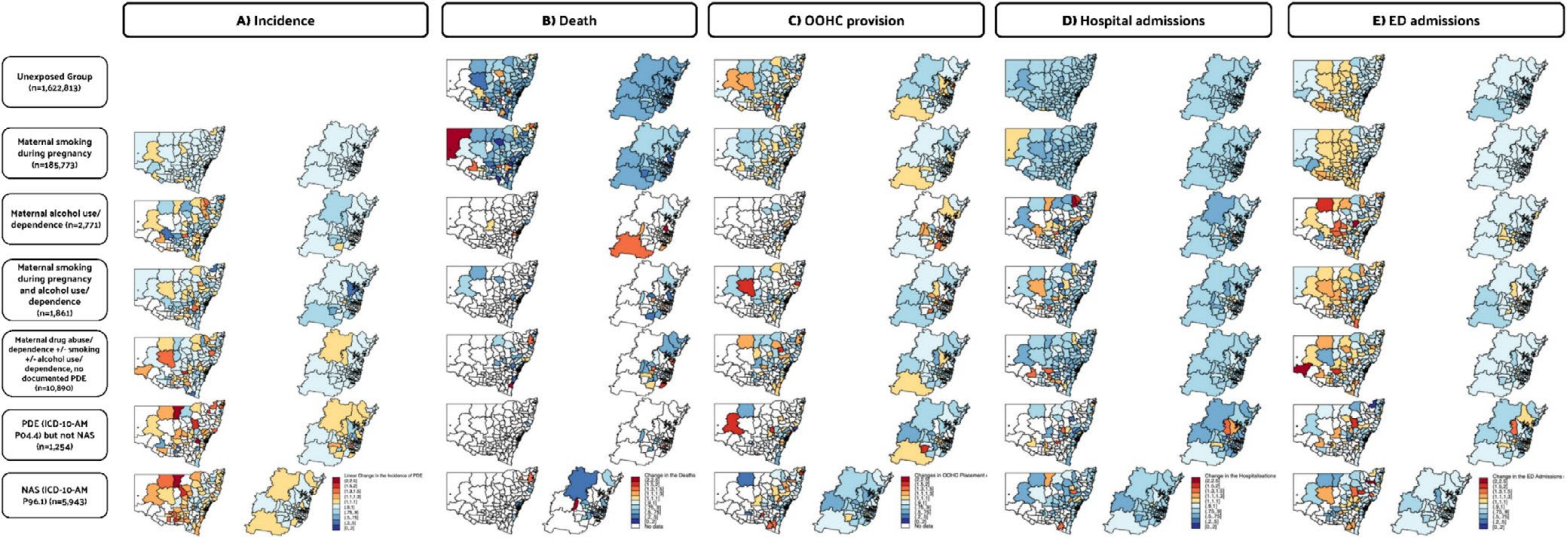
Choropleth maps demonstrating the changes in the incidence of PDE and rates of adverse childhood outcomes across NSW. (A) Incidence, (B) death, (C), OOHC provision, (D) hospital admissions and (E) ED admissions.

The temporal change in maternal substance use, PDE and NAS from 2001 to 2020 is presented in Figure 2A, with NSW on the left and Sydney on the right. Maternal smoking and alcohol use generally decreased across most areas of NSW, except for some outer regional areas where maternal alcohol use increased. Maternal exposure to illicit substances without diagnosed PDE or NAS decreased in metropolitan areas but increased in regional and remote NSW. Rates of diagnosed PDE and NAS notably increased in non‐metropolitan areas.

### 4.4 Impact of PDE on Childhood Outcomes and Temporal Changes

Cohort outcomes, including mortality, placement into OOHC, hospitalisations and ED encounters, are detailed in Table 2. The temporal changes in the incidence of these outcomes across NSW are presented in Figure 2.

#### 4.4.1 Mortality

Of the total 4046 deaths post‐discharge, 1037 (25.6%) occurred in children with PDE. Of all deaths, 45.3% occurred outside of metropolitan areas for PDE children, compared with 23.2% for children without PDE. This finding was overall not statistically significant, likely due to the relatively small number of deaths. Most deaths occurred in the first year of life. Children with a diagnosis of NAS (Group 6) were at the highest overall risk of death, while children diagnosed with PDE but not NAS (Group 5) were at the highest risk between 6 and 10 years of age (Table 2). Risk of death increased between 2001 and 2020 in children of mothers who smoked in certain regions (e.g., remote Far West, Figure 2B).

#### 4.4.2 Placement in OOHC

PDE was strongly associated with OOHC placement, particularly for infants with NAS (Group 6, Table 2). OOHC placements increased overall for regional centres between 2001 and 2020, particularly in remote and very remote areas, whereas they decreased in most metropolitan areas (Figure 2C).

#### 4.4.3 Hospitalisations and ED Encounters

PDE was primarily associated with increased risk of hospital and ED encounters throughout childhood up to 15 years of age (Table 2), especially in children with NAS between 11 and 15 years. Children with PDE also spent a greater number of days in hospital and had higher total costs of hospital admissions.

Overall risk of hospitalisations and ED encounters, however, decreased over time for children without PDE. In remote areas, hospitalisations decreased, and ED encounters increased 2.5‐fold for those with PDE (Figure 2D,E).

### 4.5 Interaction Between Geographical Remoteness and PDE

PDE and remoteness independently increased the risk of mortality, OOHC, hospitalisation and ED encounters with varying interaction effects. In their first year of life, remote infants with PDE had more hospitalisations and ED encounters compared to metropolitan infants with PDE. Between 1 and 5 years, regional children with PDE were 12% more likely to die than those living in major cities but had lower rates of hospitalisation, ED presentations and placements in OOHC. Remote children with PDE used even fewer resources but were 9% more likely to die compared to metropolitan counterparts. Children diagnosed with NAS in the neonatal period were 68% more likely to die if residing outside major cities. Effects were consistent from 1 to 15 years of age (Table 3).

**TABLE 3:** Interaction between geographical remoteness and PDE in influencing cohort outcomes.

Outcome		Effect of PDE				Effect of geographical remoteness				Interaction terms							
		Maternal smoking during pregnancy and/or alcohol use/dependence^a^ (*n* = 190 405)		Maternal drug use/dependence and/or documented PDE and/or diagnosed NAS^b^ (*n* = 18 087)		Regional (ARIA+ Categories 1 and 2) (*n* = 378 152)		Remote (ARIA+ Categories 3 and 4) (*n* = 9558)		Regional * Exposure to tobacco smoke and/or alcohol (*n* = 72 958)		Remote * Exposure to tobacco smoke and/or alcohol (*n* = 2846)		Regional * Exposure to drugs and/or documented PDE and/or diagnosed NAS (*n* = 5406)		Remote * Exposure to drugs and/or documented PDE and/or diagnosed NAS (*n* = 121)	
		aIRR^c^, ^d^ (95% CI)	*p*	aIRR^c^, ^d^ (95% CI)	*p*	aIRR^c^, ^d^ (95% CI)	*p*	aIRR^c^, ^d^ (95% CI)	*p*	aIRR^c^, ^d^ (95% CI)	*p*	aIRR^c^, ^d^ (95% CI)	*p*	aIRR^c^, ^d^ (95% CI)	*p*	aIRR^c^, ^d^ (95% CI)	*p*
Death		1.98 (1.79–2.19)	< 0.001	2.90 (2.33–3.61)	< 0.001	1.13 (1.04–1.24)	0.005	1.38 (0.91–2.11)	0.133	0.99 (0.85–1.17)	0.943	0.84 (0.46–1.54)	0.569	1.12 (0.78–1.61)	0.529	1.09 (0.25–4.72)	0.908
OOHC		12.60 (12.14–13.09)	< 0.001	52.43 (50.14–54.84)	< 0.001	1.59 (1.51–1.67)	< 0.001	1.44 (1.12–1.84)	0.004	0.51 (0.48–0.54)	< 0.001	0.36 (0.27–0.47)	< 0.001	0.41 (0.37–0.44)	< 0.001	0.38 (0.27–0.55)	< 0.001
Hospital Ad.	0–1 years	1.21 (1.18–1.23)	< 0.001	1.23 (1.17–1.30)	< 0.001	1.06 (1.04–1.08)	< 0.001	1.09 (1.01–1.17)	0.019	0.95 (0.93–0.98)	0.001	1.05 (0.94–1.17)	0.423	0.99 (0.92–1.07)	0.760	1.11 (0.70–1.77)	0.660
	1–5 years	1.06 (1.03–1.09)	< 0.001	1.22 (1.10–1.36)	< 0.001	0.95 (0.93–0.97)	< 0.001	1.03 (0.93–1.16)	0.556	1.00 (0.96–1.04)	0.943	0.99 (0.86–1.14)	0.877	0.82 (0.72–0.94)	0.005	0.70 (0.52–0.94)	0.016
	6–10 years	1.17 (1.09–1.27)	< 0.001	1.32 (1.02–1.69)	0.032	0.91 (0.87–0.95)	< 0.001	0.91 (0.78–1.06)	0.217	0.85 (0.78–0.93)	0.001	0.81 (0.67–0.99)	0.042	0.92 (0.64–1.23)	0.560	0.77 (0.47–1.26)	0.294
	11–15 years	1.10 (1.01–1.19)	0.023	1.27 (1.05–1.55)	0.015	0.92 (0.86–0.98)	0.012	0.92 (0.71–1.19)	0.535	0.95 (0.85–1.06)	0.337	0.85 (0.62–1.15)	0.288	1.15 (0.82–1.61)	0.426	1.00 (0.62–1.59)	0.984
ED Ad.	0–1 years	1.24 (1.23–1.26)	< 0.001	1.18 (1.13–1.23)	< 0.001	1.26 (1.25–1.27)	< 0.001	0.96 (0.90–1.02)	0.186	0.96 (0.95–0.98)	< 0.001	0.88 (0.79–0.99)	0.031	0.95 (0.90–1.01)	0.108	1.08 (0.73–1.59)	0.708
	1–5 years	1.12 (1.11–1.13)	< 0.001	1.06 (1.03–1.09)	< 0.001	1.33 (1.32–1.33)	< 0.001	1.06 (1.01–1.11)	0.026	1.00 (0.98–1.01)	0.778	0.86 (0.79–0.94)	0.001	0.93 (0.89–0.98)	0.003	0.66 (0.41–1.07)	0.091
	6–10 years	1.17 (1.15–1.19)	< 0.001	1.13 (1.07–1.18)	< 0.001	1.39 (1.37–1.40)	< 0.001	1.25 (1.17–1.34)	< 0.001	0.95 (0.93–0.97)	< 0.001	0.96 (0.86–1.07)	0.429	0.95 (0.88–1.02)	0.162	0.69 (0.45–1.07)	0.095
	11–15 years	1.36 (1.33–1.40)	< 0.001	1.68 (1.52–1.85)	< 0.001	1.39 (1.37–1.42)	< 0.001	1.46 (1.36–1.57)	< 0.001	0.91 (0.87–0.94)	< 0.001	0.87 (0.77–0.98)	0.025	0.87 (0.73–1.04)	0.135	0.94 (0.62–1.43)	0.776

Abbreviations: 95% CI, 95% confidence interval; Ad., admissions; aIRR, adjusted incidence rate ratio; ARIA+, Accessibility/Remoteness Index of Australia (measure of remoteness based on road distances to service centres); ED, emergency department; HR, hazard ratio; IQR, interquartile range; LOS, length of stay; NAS, Neonatal Abstinence Syndrome; OOHC, out‐of‐home care; PDE, prenatal drug exposure.

^a^ Recorded in the last hospital admission or episode of mental health care in an ambulatory care setting prior to birth.

^b^ ICD‐10‐AM (International Classification of Diseases, Tenth Revision, Australian Modification) codes P04.4 for PDE and P96.1 for NAS.

^c^ Risk compared to Unexposed Group (children with no maternal smoking during pregnancy, no maternal alcohol use/dependence, no maternal drug use/dependence, no PDE and no NAS).

^d^ Adjusted for: aboriginal and/or Torres Strait Islander mother; serious mental illness in the mother; Socio‐Economic Indexes for Areas (SEIFA) (a measure of social disadvantage in a small contained geographical area); young mother (aged < 20 years); calendar year.

## 5 Discussion

This population‐based cohort study is the first report of geographical disparities in the long‐term outcomes of PDE at a population level. Results confirm the association between PDE and adverse health outcomes [5, 9] and highlight that a growing proportion of children with PDE are from regional and remote areas, where they access fewer resources and are more likely to die.

In keeping with existing literature, children from remote areas are more likely to be affected by PDE [16, 20, 33], where they face poor access to healthcare services [5, 9, 16, 17], lower health literacy [21, 22, 23] and cultural barriers [24, 25]. The underdiagnosis of NAS observed in our rural cohort is consistent with previous reports [27, 28], which attribute this to limited antenatal screening [9] and fear of child removal [34], especially in First Nations communities [35], potentially delaying intervention and support [36].

Children with NAS are known to have increased morbidity and mortality in infancy and early childhood [5, 6, 14, 15, 37, 38]. Our study extends this to children with PDE, even without a diagnosis of NAS, and demonstrates this disparity persists into adolescence. The reasons for these adverse outcomes are multifactorial. Indeed, mothers in our PDE cohort were more likely to be younger [8, 9, 10], First Nations [5], lacking education [8, 10], unemployed [10], lower in socioeconomic status [5, 8, 9, 10] and more likely to live in unsafe, abusive circumstances [11], all aligning with prior observational studies and representing risk factors for childhood harm [39].

Temporal trends over the study period may offer insight into the efficacy of SUPS programs and rural funding initiatives, though whether services for children with PDE truly reach those in remote communities remains uncertain [40]. Our maps broadly demonstrate decreased rates of adverse outcomes for children with PDE in most metropolitan regions, while ED presentations increased in many regional and remote areas. The reasons for these findings cannot be speculated from administrative data and require further study.

Existing literature stratifying outcomes of PDE by remoteness is largely constrained to infancy and supports our finding of increased healthcare utilisation among rural infants with NAS [7, 33]. However, beyond infancy, regional children with PDE had lower rates of hospitalisation, ED presentations and placements in OOHC compared to metropolitan counterparts, yet were more likely to die. This novel finding may indicate that reduced healthcare utilisation in later childhood does not reflect lesser need in rural areas, but rather unmet need. It may also suggest that loss to follow‐up, a well‐recognised challenge in studying PDE populations that is inherently difficult to capture in longitudinal studies, disproportionately affects rural populations.

There are several strengths to our study. We demonstrate outcomes for children even without a diagnosis of PDE or NAS. Our extensive cohort allowed stratification of exposure groups, including to licit substances such as tobacco and alcohol [35].

This study has limitations. The probabilistic methods of data linkage carry a small risk of incorrect linkages (approximately 0.5%). Participants born later in the study period had less follow‐up information, and the depth of such follow‐up was highly dependent on the amount of contact with child protection and inpatient health services. Drug use may be under‐reported. Participants may not remain within their place of birth. The diagnosis of PDE may be under‐reported. While NAS is well recorded because these newborns receive a standardised treatment intervention, this may not be the case for PDE without NAS, as NAS usually refers to opiate withdrawal. This would lead to conservative reporting of effect sizes. There may be a cohort time effect over 20 years, which might not have been accurately adjusted for using calendar year. Interventions for PDE and children with PDE may have occurred at different times over different parts of the state. It is difficult to estimate the impact of the time‐cohort effect on these results.

## 6 Conclusion

Regional and remote areas are increasingly and disproportionately affected by PDE, where children use less health and social services but have higher mortality, even until 15 years of age. Despite government initiatives in Australia [29], geographical disparities persist among children with PDE and may be growing. This study emphasises the need to evaluate models of care to support women and children with PDE in regional and remote areas to prevent adverse outcomes beyond the newborn period.

## Author Contributions

T.C. developed the project idea and methodology with Prof. Ju Lee Oei and Dr. Mithilesh Dronavalli, performed statistical analysis, drafted the initial manuscript, prepared and edited the final manuscript to be submitted. M.D. developed the project idea, merged and cleaned the datasets, provided statistical supervision to Ms. Colligan, assisted with statistical interpretation, revised and approved the final manuscript to be submitted. J.L.O. developed the project idea, obtained ethics approval, submitted the initial proposal to the Centre for Health and Record Linkage (CHeReL), reviewed and approved the final manuscript to be submitted, and provided editorial input on two drafts of this manuscript. J.L.O. is responsible for Indigenous oversight of the manuscript and the project. L.D. is the consumer representative. All authors (Taylor Colligan, Ju Lee Oei, Barbara Bajuk, Lucinda Burns, Kate Lawler, Hannah Uebel, John Eastwood, Andrew Page, Evelyn Lee, Lauren Dicair, Charles Green, Michelle Dickson, Mithilesh Dronavalli) approved the final manuscript as submitted and agree to be accountable for all aspects of the work.

## Funding

This work was supported by SPHERE Mindgardens Neuroscience Network, Australian Red Cross, Alpha Maxx Healthcare, NHMRC (APP1198477) and University of Sydney.

## Conflicts of Interest

The authors declare no conflicts of interest. Ju Lee Oei has received honoraria for speaking, travel and research from Mallinkrodt Inc.

## Supporting information


**Table S1:** Summary of databases in record linkage.
**Figure S1:** Directed acyclic graph for relevant confounders in regression models.

## Data Availability

The data that support the findings of this study are available from Ministry of Health, ChEREL. Restrictions apply to the availability of these data, which were used under license for this study. Data are available from https://www.cherel.org.au/ with the permission of Ministry of Health, ChEREL.
