# Supplementary material for: The Impact of Remoteness on the Outcomes of Children With Prenatal Drug Exposure: A Population‐Based Cohort Study
Source: J Paediatr Child Health. 2026 May 25;62(7):1223–34. doi: 10.1111/jpc.70438 (PMC13378313; doi:10.1111/jpc.70438)
Supplement: Supplementary file 1 — Table S1: Summary of databases in record linkage. Figure S1: Directed acyclic graph for relevant confounders in regression models. [file JPC-62-1223-s001.docx]

| **Supplementary Table 1. Summary of Databases in Record Linkage** | | |
| --- | --- | --- |
| **Database** | **Information Available** | **Dates** |
| **PRIMARY DATABASES** | | |
| **NSW Perinatal Data Collection (PDC Babies)** | - Maternal demographic information - Maternal medical information - Maternal obstetric information - Delivery information - Infant demographic information, including gestation, and birth weight, condition, APGAR score and resuscitation details | 1 Jul 2001 to  31 Dec 2020 |
| **NSW Perinatal Data Collection (PDC Mothers)** |  | 1 Jul 2001 to  31 Dec 2020 |
| **LINKED DATABASES** | | |
| **NSW Admitted Patient Data Collection (APDC)** | - Information on separations (discharges, transfers and deaths) and health service facility use for NSW residents within and outside NSW - Demographic details and administrative items - Diagnoses (ICD-10-AM) – up to 50 diagnoses associated with each episode of care (included NAS [P96·1], newborn affected by maternal drugs of addiction [P04·4], and other maternal drug/alcohol related disorders [F10-F19]). | 1 Jul 2001 to  31 Dec 2021 |
| **NSW Mental Health Ambulatory Data Collection (MH AMB)** | - Demographic information - Diagnosis (ICD-10-AM), used to identify maternal diagnoses relating to alcohol/drug abuse/dependence. | 1 Jul 2001 to  31 Dec 2021 |
| **NSW Registry for Births, Deaths & Marriages (RBDM)^a^** | - Demographic information - Date of birth and date of death (used to calculate age of death) | 1 Jul 2001 to  31 Dec 2021 |
| **NSW Cause of Death Unit Record**  **File (COD URF)** | - Demographic information - Cause of death (ICD-10-AM) | 1 Jul 2001 to  31 Dec 2020 |
| **NSW Family and Community Services Dataset – KiDS Data**  **Collection (DCJ KiDS)** | - Demographic information - Placement date, duration of care, and type of care (e.g. Foster Care, Relative/Kinship Care) | 18 Jul 2001 to  30 Jun 2021 |
| NSW, New South Wales; ICD-10-AM, International Statistical Classification of Diseases 10^th^ Edition Australian Modification;  ^a^RBDM death records with a death date after 31 Dec 2020 do not have corresponding COD URF records. | | |

Supplementary Figure 1. Directed Acyclic Graph for Relevant Confounders in Regression Models

Young mother (<20yr)

First Nations Status

Serious mental illness

Outcome: out-of-home care

Social disadvantage (SEIFA)

# Prenatal drug exposure

Time (calendar year)

Remoteness (ARIA)

Health outcomes: hospitalisations, ED visits, death
